# Estimation of linkage disequilibrium and effective population size in New Zealand sheep using three different methods to create genetic maps

**DOI:** 10.1186/s12863-017-0534-2

**Published:** 2017-07-21

**Authors:** Vincent Prieur, Shannon M. Clarke, Luiz F. Brito, John C. McEwan, Michael A. Lee, Rudiger Brauning, Ken G. Dodds, Benoît Auvray

**Affiliations:** 1 AgResearch, Invermay Agricultural Centre, Private Bag 50034, Mosgiel, 9053 New Zealand; 2 Centre for Genetic Improvement of Livestock, University of Guelph, N1G2W1, Guelph, Canada; 3 Department of Mathematics and Statistics, University of Otago, Dunedin, 9058 New Zealand; 4 Current address: France Limousin Sélection, Pôle de Lanaud, 87220 Boisseuil, France

**Keywords:** Genetic diversity, LD, Ovine 50 K SNP chip, genetic maps, *Ovis aries*

## Abstract

**Background:**

Investments in genetic selection have played a major role in the New Zealand sheep industry competitiveness. Selection may erode genetic diversity, which is a crucial factor for the success of breeding programs. Better understanding of linkage disequilibrium (LD) and ancestral effective population size (N_e_) through quantifying this diversity and comparison between populations allows for more informed decisions with regards to selective breeding taking population genetic diversity into account. The estimation of *N*_*e*_ can be determined via genetic markers and requires knowledge of genetic distances between these markers. Single nucleotide polymorphisms (SNP) data from a sample of 12,597 New Zealand crossbred and purebred sheep genotyped with the Illumina Ovine SNP50 BeadChip was used to perform a genome-wide scan of LD and *N*_*e*_. Three methods to estimate genetic distances were investigated: 1) M1: a ratio fixed across the whole genome of one Megabase per centiMorgan; 2) M2: the ratios of genetic distance (using M3, below) over physical distance fixed for each chromosome; and, 3) M3: a genetic map of inter-SNP distances estimated using CRIMAP software (v2.503).

**Results:**

The estimates obtained with M2 and M3 showed much less variability between autosomes than those with M1, which tended to give lower *N*_*e*_ results and higher LD decay. The results suggest that *N*_*e*_ has decreased since the development of sheep breeds in Europe and this reduction in N_e_ has been accelerated in the last three decades. The *N*_*e*_ estimated for five generations in the past ranged from 71 to 237 for Texel and Romney breeds, respectively. A low level of genetic kinship and inbreeding was estimated in those breeds suggesting avoidance of mating close relatives.

**Conclusions:**

M3 was considered the most accurate method to create genetic maps for the estimation of LD and N_e_. The findings of this study highlight the history of genetic selection in New Zealand crossbred and purebred sheep and these results will be very useful to understand genetic diversity of the population with respect to genetic selection. In addition, it will help geneticists to identify genomic regions which have been preferentially selected within a variety of breeds and populations.

**Electronic supplementary material:**

The online version of this article (doi:10.1186/s12863-017-0534-2) contains supplementary material, which is available to authorized users.

## Background

Sheep were domesticated around 9000 years ago during the Mesolithic period in the Fertile Crescent region (Middle East, Asia). Since domestication, sheep have spread throughout the whole world and have been raised and genetically improved for various purposes such as meat, wool, fiber, skin and milk [1, 2]. The footprints of this selection remain in the sheep genome and can be used to investigate the history of the species and its spread across the world [3, 4]. Natural selection combined with recent breed creation has led to an overall decrease of the genetic diversity observed within isolated populations [5]. Recently, this reduction in genetic diversity has been accelerated by intensive selection coupled with creation of breeds derived from small founder populations.

The introduction of sheep in New Zealand (NZ) commenced 200 years ago. The genetic gain resulting from breeding programs has resulted in significantly increased efficiency of sheep production. However, the management of genetic variability between and within breeds is one of the major challenges that will determine the success of future selection [6]. Therefore, it is crucial to understand the genetic structure of the selected populations and to assess its genetic diversity. This can be achieved using genomic information and for this purpose, we utilized a dataset of 12,597 animals genotyped with the Illumina OvineSNP50 BeadChip; a SNP array developed by the International Sheep Genomics Consortium (ISGC) in conjunction with Illumina [4, 7].

Estimating the effective population size (*N*_*e*_) [8] is a useful way to evaluate the loss or gain of diversity across time and allows identification of selection events, such as bottlenecks [9]. It is possible to estimate *N*_*e*_ from molecular data using the *r* ^2^ coefficient [10], which is a measure of linkage disequilibrium (LD). LD studies are often used to illustrate the genetic relationships within and between different populations [11, 12]. Levels of LD are also an important parameter for the implementation of genomic selection [13] in livestock species, including sheep [14, 15]. LD estimated from microsatellites, variable number tandem repeats (VNTR) or single nucleotide polymorphisms (SNP) markers reflect the footprints of past genetic events [16, 17]. Several studies on farmed species have been performed to understand the population structure of purebred and crossbred animals in species such as beef cattle [18]. In sheep, different studies which use genetic markers have been published [19–21]. However, there is still a lack of information for NZ sheep breeds, crossbred and composite populations. Therefore, the main goal of this study was to compare estimates of LD and *N*_*e*_ over time, using the Illumina OvineSNP50 BeadChip SNP array and three different methods to estimate genetic distances between SNP markers. The study focused on four common NZ sheep breeds (Coopworth, Romney, Perendale and Texel) and three composite breed groupings, as described by Dodds et al. [22].

## Methods

### Animals

A total of 12,597 genotyped animals from research and ram breeders’ flocks involved in the Ovita R&D program [23] were used in this study. The sex ratio was 70% males and the majority of these individuals had recorded pedigree. Seven breed groups were included in the analysis, in which four purebred groups were defined as having at least 75% of the predominant breed: Coopworth (Coop), Romney (Rom), Perendale (Peren) or Texel (Tex), and three composite groups (as described in Dodds et al. [22] and Table 1). The composite groups were: CompRCP, which includes animals with more than 50% of (Rom + Coop + Peren) and less than 25% of Tex; CompRCPT, with more than 50% of (Rom + Coop + Peren) and more than 25% Tex; and Comprcp2 which includes a proportion of (Rom + Coop + Peren) greater than 30% and lower than 50%. Breed groups were assigned using Sheep Improvement Limited (SIL, http://www.sil.co.nz/) recorded breed. When the recorded breed included a composite breed containing a known proportion of Romney, Coopworth, Perendale or Texel, it was replaced using those proportions so that animals subsequently meeting a composite group definition were included in the analysis. The dataset description, which includes the number and breed proportion of each pure and crossbred animal in the dataset is shown in Table 1. Pedigree information for all the animals was available from SIL and a description of the pedigree information is shown in Additional file 1.

**Table 1:** Animal and SNP data set description

Breed group	Breed definition	Markers SNPs		Animals	
		Before QC	After QC	Before QC	After QC
Rom	Rom ≥0.75	48,277	41,505	5908	5820
Coop	Coop ≥0.75		43,821	2298	2269
Peren	Peren ≥0.75		44,365	794	777
Tex	Tex ≥0.75		42,970	414	409
CompRCP	Not in the above, Rom + Coop + Peren >0.5 AND Tex <0.25		44,880	1179	1161
CompRCPT	Not in the above, Rom + Coop + Peren >0.5 AND Tex ≥0.25		44,654	379	375
Compcrp	Not in the above, 0.3 < Rom + Coop + Peren ≤0.5		45,572	1625	1603
Total - Separated by breed				12,597	12,414
All breeds			43,222	12,597	12,518

*Rom* Romney breed, *Coop* Coopworth breed, *Peren* Perendale breed, *Tex* Texel breed, *QC* quality control, *SNPs* Single Nucleotide Polymorphisms

### Data quality control (QC)

The genomic data was pre-processed using a previously described pipeline [24]. In brief, this process included a filter on SNP call-rate, quality score from the Illumina scoring algorithm [25], minor allele frequency (MAF > 0) and extreme departure from Hardy Weinberg Equilibrium (HWE, *p* ≤ 10^−6^). Following QC, 48,277 SNPs from a total of 53,903 SNPs remained for further analysis. The same steps were subsequently applied for each breed. Sex was checked for possible errors using non-pseudo-autosomal X chromosome genotypes. If more than 3% of SNPs located on the X chromosome (including pseudo-autosomal markers) were heterozygous, the sex of the individual was considered as female, otherwise it was considered male. Next, 1161 non-autosomal SNP markers were removed. Animals with a low call-rate across SNPs (≤0.95) and pairs of animals with high identity by state (IBS) genotypes based on 2000 autosomal markers (≥0.95) were also removed from the analysis, as they could be duplicated samples or monozygotic siblings (e.g. identical twins or triplets). This dataset was used for investigation of MAF, heterozygosity, kinship between breeds and estimation of genomic inbreeding. Prior to the LD analysis, SNPs with a MAF lower than 0.05 were removed. Different sets of SNPs were removed depending on the breed. The proportions of SNPs within categories of MAF are presented in Table 2. In parallel, a second set of QC was performed on the full dataset (all breeds combined). Threshold settings for those QC were the same as those applied for the single breed process, with the exception that the HWE test was not performed, because applying HWE test on a population composed of an ensemble of genetically distinct populations would lead to inappropriate removal of markers. Instead, we only removed SNPs that did not pass the HWE test in any of the seven breeds, in the “breed by breed” QC workflow (Additional file 2). R version 3.0.1 software [26] including the GenABEL R package (version 1.7–3) [27] was used for the statistical analysis.

**Table 2:** Mean heterozygosity, average inbreeding per breed, average genomic relatedness between animals between and within breed estimated from a genomic relationship matrix and minor allele frequencies

Item		Breed group						
		Rom	Coop	Peren	Tex	CompRCP	CompRCPT	Comprcp2
Relationship coefficient between animals	Rom	0.000						
	Coop	0.000	0.011					
	Peren	0.000	0.000	−0.001				
	Tex	0.000	0.000	0.000	0.008			
	CompRCP	0.000	0.009	0.000	0.001	0.015		
	CompRCPT	0.000	0.007	0.000	0.003	0.011	0.018	
	Comprcp2	0.001	0.003	0.000	0.002	0.007	0.006	0.063
Inbreeding	_	0.030	0.020	0.002	0.007	0.010	−0.007	0.092
Heterozygosity	_	0.358	0.351	0.376	0.354	0.381	0.377	0.370
MAF in % of SNP per categories	MAF ≤ 0.01	1.9	1.8	1.3	1.9	0.9	1.1	0.6
	0.01 < MAF ≤ 0.05	5.2	4.7	4.1	5.8	3.7	3.6	2.5
	0.05 < MAF ≤ 0.1	7.1	7.1	6.3	7.6	6.0	6.1	5.4
	0.1 < MAF ≤ 0.2	17.5	17.4	17.3	17.6	17.0	16.8	16.7
	0.2 < MAF ≤ 0.3	20.9	20.9	21.6	20.7	21.5	22.0	22.1
	0.3 < MAF ≤ 0.4	22.9	23.4	23.9	22.8	24.7	24.4	25.7
	0.4 < MAF ≤ 0.5	24.6	24.7	25.5	23.5	26.3	26.0	26.9

### Genetic diversity

Average heterozygosity, genomic kinship and inbreeding coefficients were estimated to quantify the genetic diversity of the populations under investigation. The average heterozygosity (*H*_*e*_) was assessed before the estimation of the missing genotypes which tend to overestimate *H*_*e*_. Individual *H*_*e*_, based on SNPs which passed the first set of QC for a given breed, was calculated and used to estimate breed *H*_*e*_ (Additional file 2). Genomic relationship between breeds was estimated as described in Auvray et al. [23]. A genomic relationship matrix (**GRM**) accounting for breed composition (using breed proportions of “Rom”, “Coop”, “Peren”, “Tex”, and “Others” in each animal) was calculated [22, 23]. The mean relationship within and between the seven breed groups was calculated and the mean of the **GRM** diagonal elements minus 1 was considered as the average inbreeding coefficient per breed group (Table 2).

### Genetic relatedness between individuals

To understand the genetic relationships between animals within and across breed groups, matrices of genetic distances (**D**) between the animals were computed from the **GRM** after the estimation of missing genotypes, as *d*_*ij*_ = 1 − *g*_*ij*_ if *i* ≠ *j* and *d*_*ij*_ = 0 if *i* = *j* (where *d*_*ij*_ and *g*_*ij*_ are the elements of **D** and **GRM**, respectively, for animals *i* and *j*. Multi-dimensional scaling (MDS) was used to visualize genetic distances between animals for the entire dataset and for each breed separately. The 1% most genetically distant individuals within breed (calculated using the sum of squared distances from all the others animals in the same breed) were removed from the dataset before further analyses, as these genotypes were assumed to be errors of parentage or breed recording. For each breed, the two largest flocks were used to investigate potential sub-populations that could exist within a single breed.

### Genetic maps and linkage analysis

The estimation of genetic distances was performed using CRIMAP software [28] (v2.503, modified by J.F Maddox) on the International Mapping Flock (IMF). The IMF is a flock including Texel, Merino, Perendale, Romney and Coopworth sheep breeds over three generations and nine full-sib families, for 127 genotyped animals [29]. Genetic distances were previously estimated using the IMF with 1000 microsatellites markers [30]. For the present study, a subset of SNPs approximately evenly spaced along the whole genome was selected. Two SNPs per centiMorgan (cM) were chosen according to their position on the genetic map of Churra sheep [31]. This SNP distance is about the best resolution that can be achieved with the IMF resource and was assumed to be sufficient for detecting recombination rate changes across a chromosome. This resulted in a subset of 6689 SNPs covering 95% of the sheep genome. Only SNPs located on the same chromosome in both versions 2 and 3 of the sheep genome assembly (6629 SNPs) were kept for further analysis. Finally, 6448 SNPs remained after the first set of QC in the “breed by breed” workflow for the genetic distance estimation. The SNPs had an average of 75.0 informative meiosis, with an average of 25.8 phase known informative meiosis (Additional file 3). There were between 113 (OAR 24) and 675 (OAR1) SNPs used per chromosome. The SNP order was taken from version 3 of the sheep genome assembly and assumed to be the true order. The distances between adjacent SNPs were as the sex-averaged Kosambi distances from running the CRIMAP fixed option using all SNPs on a chromosome.

Three methods to convert physical distances to genetic distances were investigated:1) M1: using the “usual” conversion ratio of 1 cM per Mb across the whole genome [31]; 2) M2: using a chromosome specific ratio of genetic distance over physical distance (Table 3); and, 3) M3: using estimates of genetic position for every SNP. The chromosome specific ratios of genetic over physical distance (used for M2) were obtained by dividing the sum of genetic distances on a chromosome (in cM) by the physical length (in Mb). For M3, the ratios of genetic to physical distances were estimated for each interval on the map. To avoid overestimation, intervals with extreme ratios (≥99.9 percentile) were removed. Next, the local polynomial fitting method, implemented by the R function *loess* [26] was used to regress the genetic positions of the 6448 SNPs on their physical positions, and to interpolate the genetic positions of the entire Illumina OvineSNP50 BeadChip SNP dataset. The ratio ∆cM/∆Mb indicates the variation of the estimated recombination rate for each interval between two SNPs and measures the amount of recombination that takes place within a given physical distance on the genome. For a detailed discussion about recombination rate, please see e.g., Jensen-Seaman et al. [32]. For a few intervals, a negative ∆cM was observed, and these intervals were removed as they imply an alternative marker order. Negative values can be obtained from incorrect SNP positions on the chromosome, or from smoothing artefacts. The correlation between the average ratio of recombination per chromosome and the chromosome length was also calculated.

**Table 3:** Genetic distances estimated with CRIMAP for a data set of 6448 SNPs

Chromosome	Ovine Genome V3.1. Physical size (Mb)	N. of SNPs	Genetic size in cM (Maddox [30])	Genetic size in cM (estimated)	cM/Mb
OAR1	275	675	341	323	1.17
OAR2	249	598	308	292	1.17
OAR3	224	575	321	273	1.22
OAR4	119	321	129	155	1.30
OAR5	107	285	153	152	1.43
OAR6	116	303	155	144	1.24
OAR7	100	281	134	137	1.37
OAR8	90	243	125	124	1.38
OAR9	94	261	126	126	1.34
OAR10	86	202	100	116	1.34
OAR11	62	173	119	110	1.78
OAR12	77	206	94	112	1.45
OAR13	83	241	132	130	1.56
OAR14	62	167	116	118	1.90
OAR15	80	190	109	110	1.37
OAR16	71	205	81	87	1.22
OAR17	72	196	121	114	1.59
OAR18	69	187	120	115	1.68
OAR19	60	177	75	108	1.81
OAR20	51	159	81	86	1.70
OAR21	49	113	74	79	1.60
OAR22	50	149	85	83	1.67
OAR23	62	160	76	90	1.45
OAR24	42	113	89	84	2.01
OAR25	45	133	69	77	1.72
OAR26	44	135	70	76	1.75
TOTAL	2439	6448	3403	3421	1.51

The physical size in Mb of a chromosome is given by the last SNP position on the Ovine Genome V3.1. The ratio cM/Mb represents the updated genetic size in cM, estimated per chromosome, divided by the size in Mb. Correlation between the physical size per chromosome and the ratio cM/Mb is strongly negative, −0.7

### Estimation of the extent of LD

Studies of N_e_ history are commonly based on *r* ^2^, a measure of LD [33]. Let *D* be the difference between the haplotype frequencies observed and those expected under the hypothesis of independence between alleles = ƒ(***AB***) – ƒ(***A***)ƒ(***B***), where ƒ(***AB***) is the observed haplotype frequency and ƒ(***A***) and ƒ(***B***) the observed allele frequencies for allele A at one locus and allele B at the other locus. The *r* ^2^ measure, which is the square of the correlation between alleles at loci A and B (scored as 0 and 1) was calculated as:${r}^2=\frac{{\left[ f\left(\boldsymbol{A}\boldsymbol{B}\right)- f\left(\boldsymbol{A}\right) f\left(\boldsymbol{B}\right)\right]}^2}{f\left(\boldsymbol{A}\right) f\left(\boldsymbol{a}\right) f\left(\boldsymbol{B}\right) f\left(\boldsymbol{b}\right)}$. Values of *r* ^2^ range from zero to one (when the two alleles at the first locus are found with a unique allele at the second). For bi-allelic markers, *r* ^2^ is commonly used as a measure of LD because it has a known χ^2^ distribution under the null hypothesis of linkage equilibrium [34]. Consequently, *r* ^2^ values were computed for each pair of SNPs on each chromosome using the function *r2fast* from the GenABEL R package [35]. The LD decay curve was obtained using a local polynomial regression of observed pairwise *r* ^2^ values on genomic distances for a randomly selected sample of 20,000 marker pairs for each chromosome. This process was repeated 30 times to avoid sampling artefacts, and means and variances over repetitions and chromosomes at 2000 values equally spaced between 0.5 cM to 50 cM were estimated. Finally, the LD decay per breed was considered as the mean of the expected *r* ^2^ (from the local polynomial regression) over each of the 26 chromosomes, estimated per chromosome at each of the 2000 genetic distances, weighted by the inverse of the variances previously estimated on the 30 repetitions.

### Estimation of effective population size over generations

The equation used to estimate *N*_*e*_ over time was: ${N}_e=\frac{1}{4 c}\left(\frac{1}{E\left({r}^2\right)}-1\right)$ [36], where *c*, the genetic distance in Morgans, was estimated for each chromosome in the linkage analysis and *E*(*r* ^2^) is the expected *r* ^2^ for the distance *c*. Time, expressed in generations was calculated as: $T=\frac{1}{2 c}$ [10]. *N*_*e*_ was estimated for each chromosome and generation in the past starting at one generation (at a distance of 50 cM), and up to 100 generations in the past (at a distance of 0.5 cM). Overall *N*_*e*_ was calculated as the average *N*_*e*_ for the 26 autosomal chromosomes weighted by the inverse of the variances per chromosomes, as was done for LD decay. For both LD and *N*_*e*,_ a Shapiro-Wilk test [37] was used to check if the estimations at each of the 2000 points on the 26 chromosomes were normally distributed around the mean, using a Bonferroni corrected α level (≤0.05/2,000), and no evidence for non-normality was observed. The 95% confidence interval based on the means and variances across chromosomes was computed for the *r* ^2^ and *N*_*e*_ values obtained with the three methods. The differences between weighted variances and between weighted means at each position using the different methods were investigated using a parametric bootstrapping process. A total of 1000 bootstraps were performed for each position under the assumption of a normal distribution, and both null hypotheses *H*_0_ : *σ* ^2^(*M*1) = *σ* ^2^(*M*2) and *H*_0_ : *μ*(*M*1) = *μ*(*M*2) were tested considering ***α*** = **0.05**. The same procedure was used to compare M1 to M3 and M2 to M3.

## Results

### Genotypes analysis

The expected SNP positions in cM and the expected recombination rates for the entire sheep genome are presented in Additional files 4 and 5. These files can be loaded directly into the UCSC genome browser following instructions at: http://genome.ucsc.edu/goldenPath/help/hgTracksHelp.html#ADD_CT. They should be used with version 3.1 of the sheep genome assembly: Aug. 2012 (ISGC Oar_v3.1/oviAri3).

### Genetic diversity

The estimation of genomic kinship within and between breeds, average genomic inbreeding, overall heterozygosity per breed and the MAF distributions over the genome are presented in Table 2. The genomic inbreeding levels ranged from −0.007 to 0.092 and the average heterozygosity ranged from 0.351 to 0.381 (Table 2). The genomic kinship within and between breeds and the estimation of the average genomic inbreeding within a given breed revealed that the animals are mostly not genetically related. The average heterozygosity per breed suggests that the genetic diversity is similar for all purebred animals, with a slightly greater value for the Perendale breed. As expected, the three composite breeds presented a greater level of heterozygosity. All breed groups included in this investigation presented a proportion of SNPs with high polymorphism (MAF ≥ 0.30) greater than 45%.

#### Population description based on pedigree information

A complete description of the populations included in these study was also done based on pedigree information. In summary, the number of animals in the pedigree ranged from 379 (CompRCPT) to 5908 (Rom). The average pedigree depth for all the populations was 5.64 and 948 animals had pedigree depth equal to zero, which was a limitation for the calculation of genetic diversity metrics such as N_e_, as will be discussed later. The average relatedness between animals was 0.003 and the average inbreeding was 0.012. Other pedigree statistics are shown in Additional file 1.

### Relatedness between individuals and evidence for breed differentiation and admixture

**Fig. 1:**
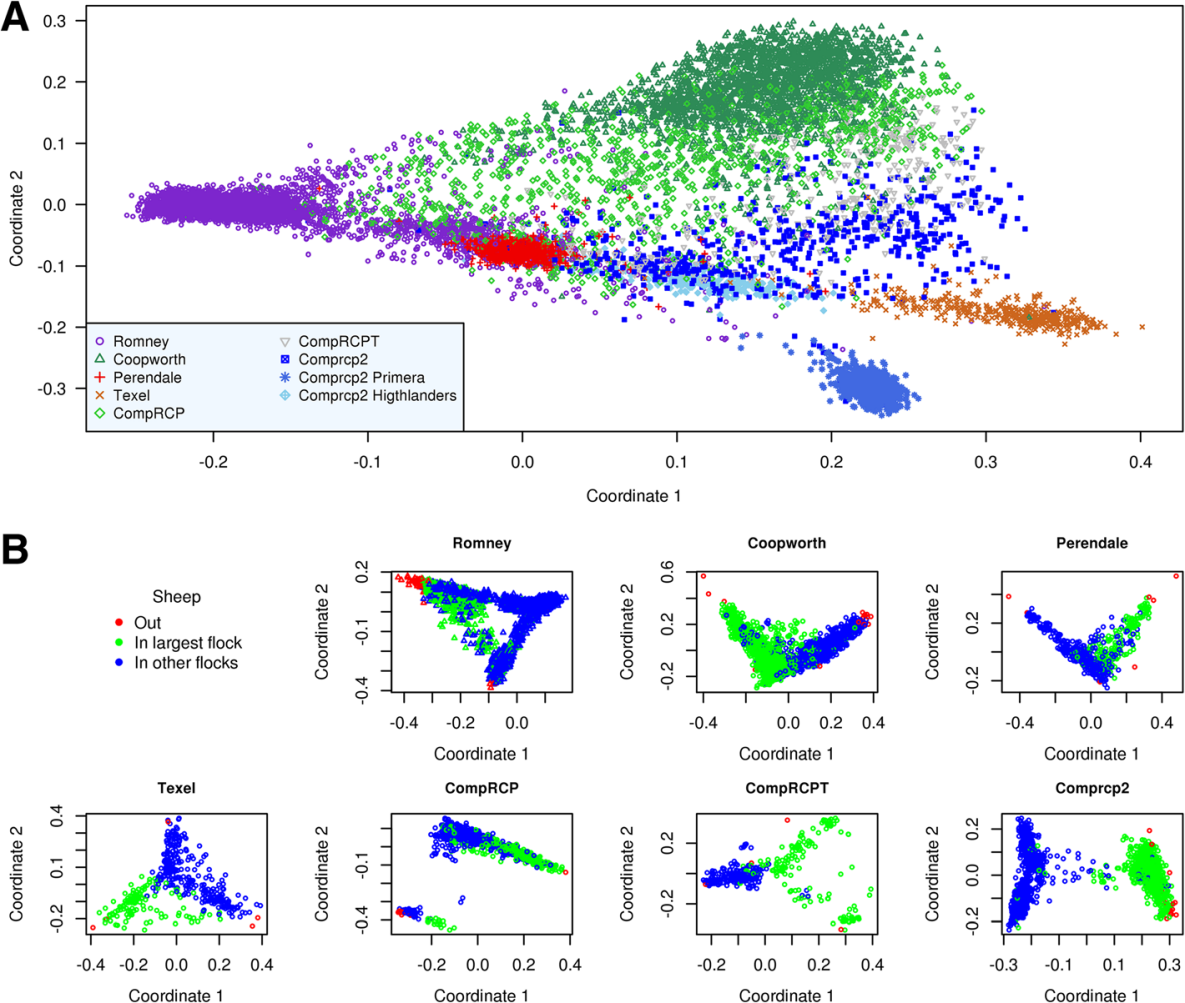
**a** Multi-Dimensional Scaling plot of genetic distances between animals. Multi-breed representation. **b** Multi-Dimensional Scaling of genetics distances. Single breed representation

The genetic structure between and within breed were investigated by MDS using a matrix of genetic distances between animals (Fig. 1a, b). The results clearly represented the expected relationship between breeds and it was consistent with the breeds’ development history. The multi-breed plot (Fig. 1a), mainly highlights the genetic segregation or relationship between breeds, while the single breed plots (Fig. 1b) show that breeds are in fact composed of distinct sub-groups. The four pure breeds are well differentiated and the composites are located as expected at variable distances between the pure breed clusters (Fig. 1a). One private flock is differentiated in Romney sheep and two commercial populations managed by private companies for Comprcp2 are also clearly separated. It is interesting to note that the two most distant breeds on the first axis of Fig. 1a are Texel and Romney. The 1% most distant sheep were removed from further analysis and are represented by the red dots (‘out’). The results for the different pure and crossbred animals based on the first two coordinates of the MDS are consistent with what was expected according to breed predictions. All the breed groups appeared clearly composed of two (Comprcp2, CompRCP, Coopworth and Perendale) or three subgroups (CompRCTP, Romney and Texel). The relative genetic diversity observed is also consistent with the results of MAF, average heterozygosity, genetic kinship and genomic inbreeding (Table 2).

### Genetic map

**Fig. 2:**
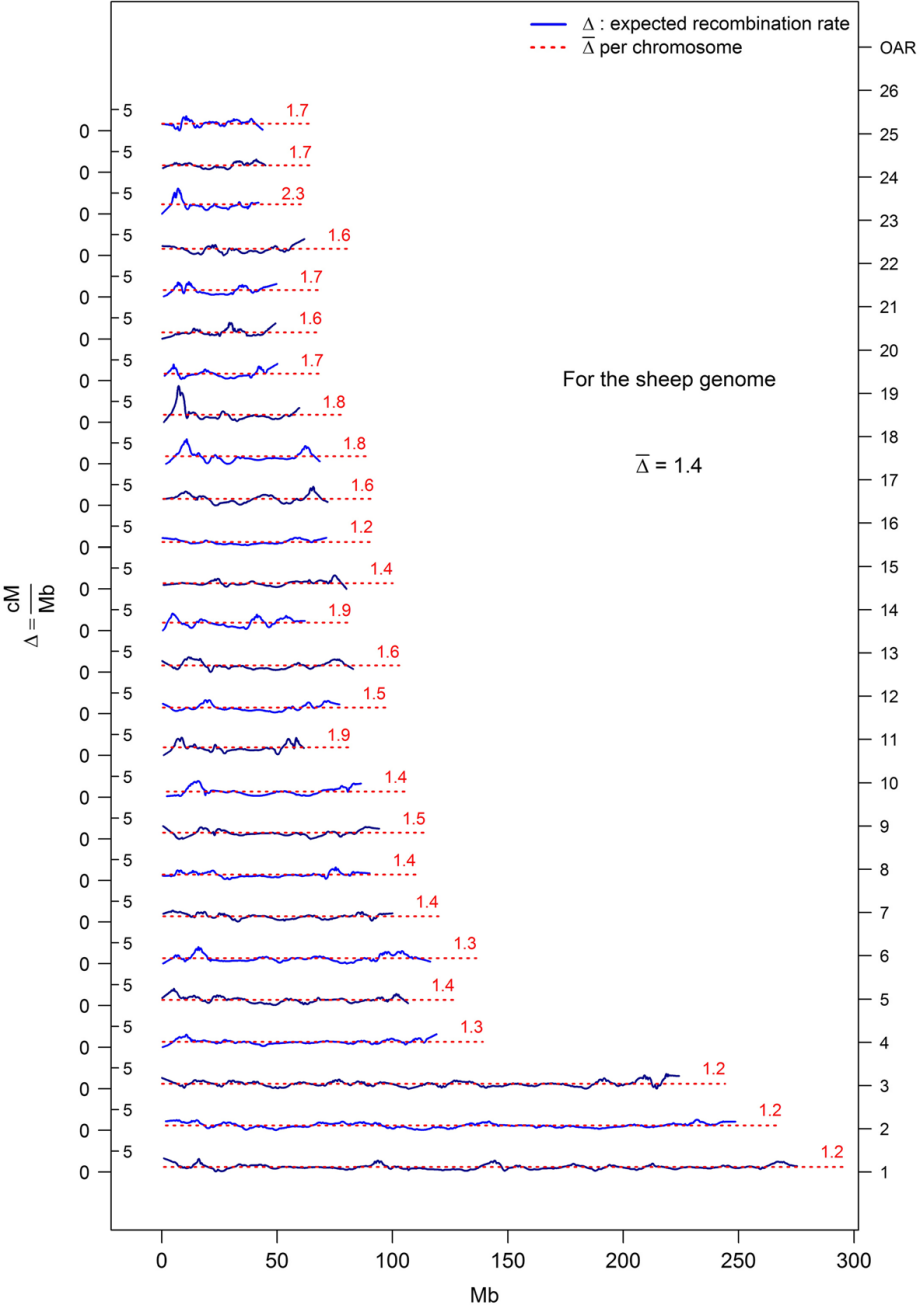
Recombination rate expressed in centiMorgan per Megabase for the sheep genome assembly version 3

The correlation between the ratio cM/Mb and the chromosome length (in Mb) was negative (−0.7, *P* = 6.8 x 10^−5^, Table 3), indicating that the ratio of recombination events is greater for shorter chromosomes. As shown on Fig. 2, which presents *loess* estimates of recombination rates (cM/Mb) along the genome, recombination rates are quite variable, in contrast to the assumption made by methods M1 and M2. Figure 2 also suggests that the recombination rates tend to be more variable on short chromosomes.

### Estimations of extent of LD and ancestral effective population size

**Fig. 3:**
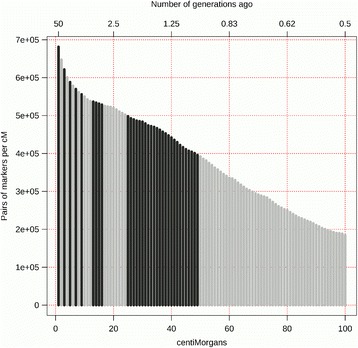
Number of SNP pairs per centiMorgan and relation between genetic distances and the time expressed in generations in the past

Levels of LD varied between and within breed groups. In addition, they varied according to chromosome and SNP marker position on the chromosome. The differences between the three curves can only be observed when enough pairs of markers are available for each generation in the past. For this purpose, the number of SNP pairs per generations was investigated and represented graphically in Fig. 3. Additional file 6 gives the number of SNP pairs per generation. The number of SNP pairs available on average per breed group (after QC) decreased exponentially from 11,181,609 between the first and the second generation to 3426 between the 99th and the 100th generations. Moreover, the average distance between markers using the Illumina Ovine SNP50 BeadChip was 50 Kb. This distance corresponds to approximately 0.5 cM (using M1), which corresponds to 100 generations in the past. Consequently, studying the evolution of *N*_*e*_ across time with the Illumina Ovine SNP50 BeadChip was only possible up to 100 generations in the past. In addition, when using the other methods (M2 or M3), only marker pairs with a recombination rate of 1 cM/Mb or less (Fig. 2) could be used to investigate *N*_*e*_ at 100 or more generations in the past (assuming they were evenly spaced). Thus, investigating *N*_*e*_ in older generations leads to very little variation between the three methods, but this would probably not be the case using a denser SNP chip panel. Alternatively, for distances between markers greater than 10 cM (five generations ago or less), differences observed between the three methods for *r* ^2^ and *N*_*e*_ were small, but still significant. A higher density SNP chip panel or even sequencing data would provide more information to estimate *N*_*e*_ in more distant generations in the past. However, the main focus of this study was to investigate *N*_*e*_ in more recent generations, which has important implications for breeding programs.

**Fig. 4:**
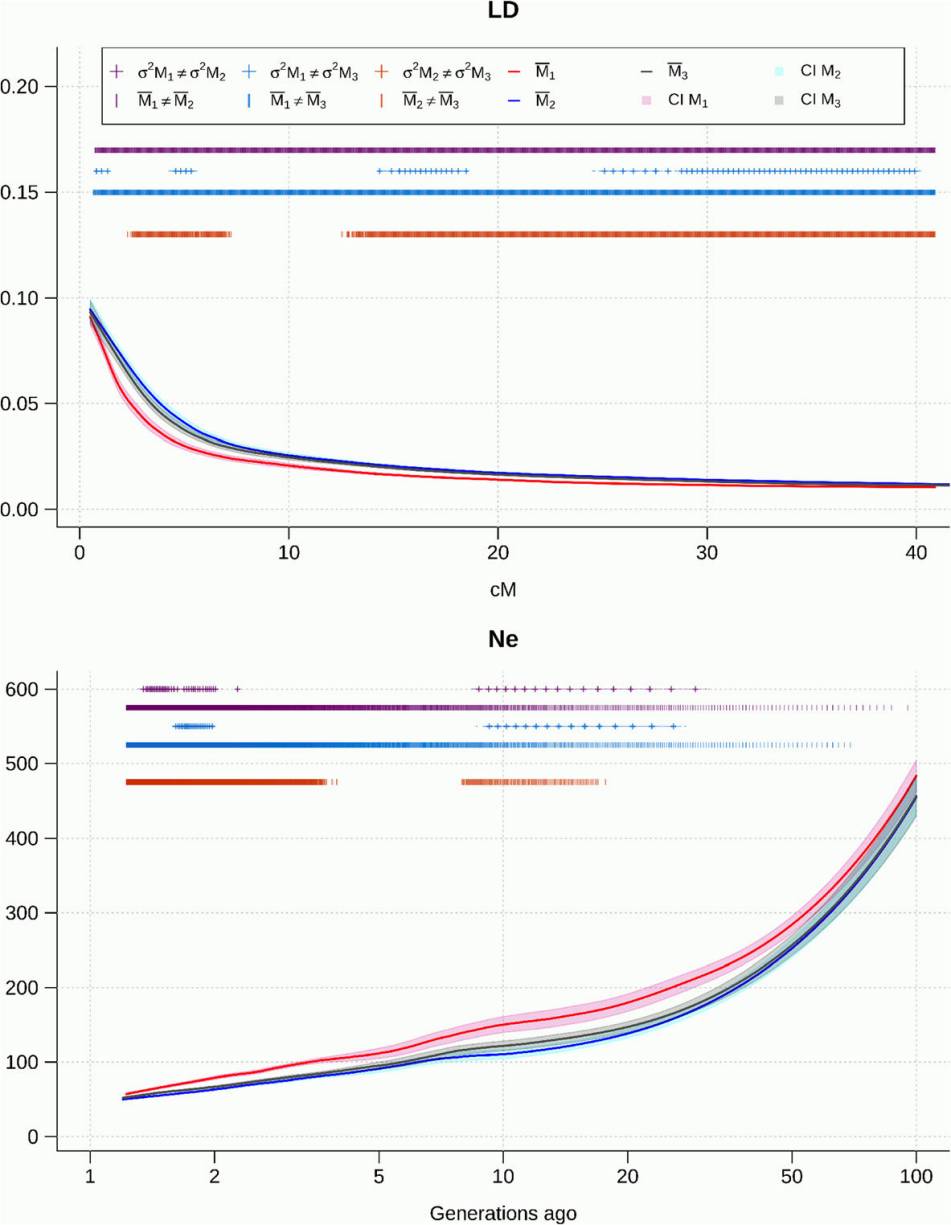
Linkage disequilibrium (LD) decay over genetic distances and evolution of *N*_*e*_ over time estimated using the three methods in Coopworth sheep

**Fig. 5:**
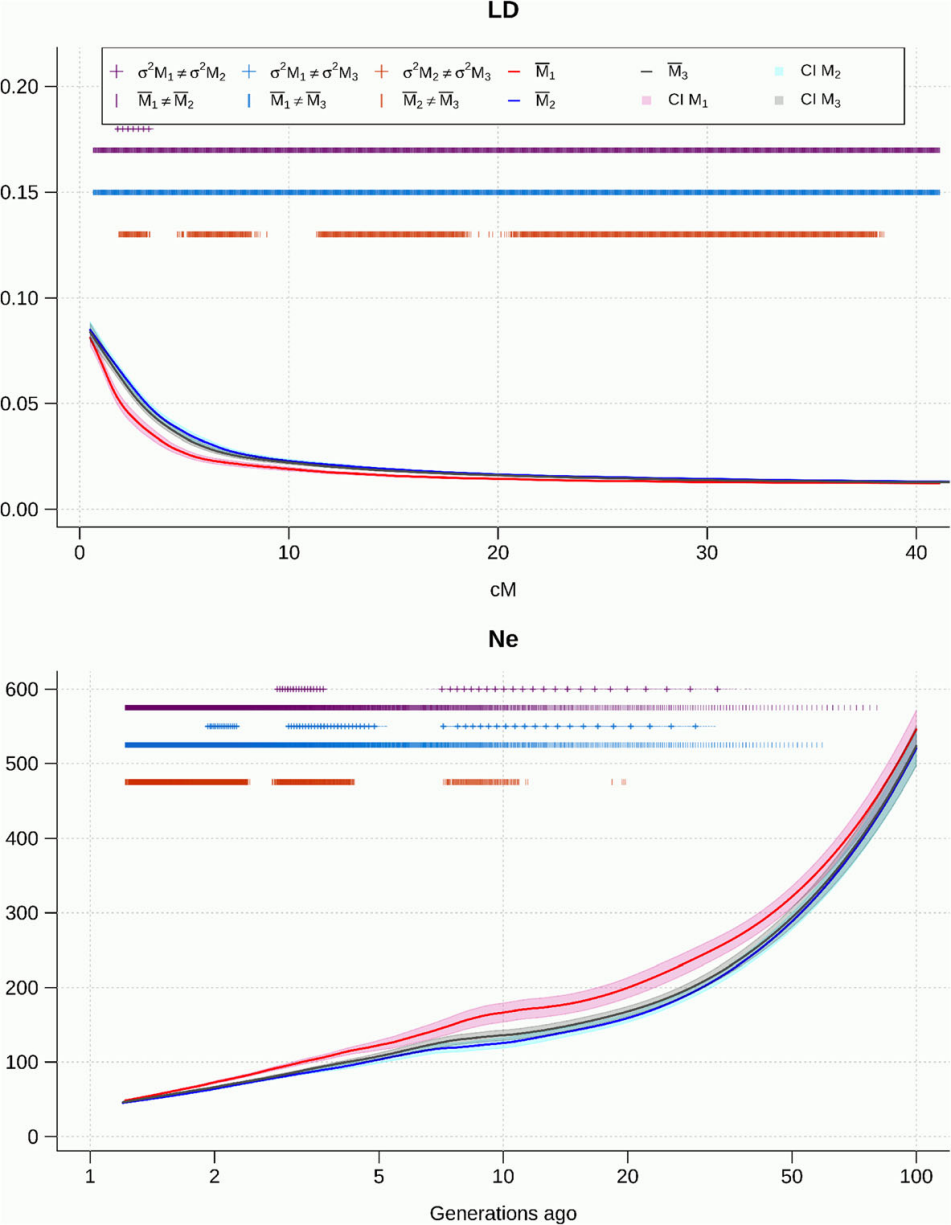
Linkage disequilibrium (LD) decay over genetic distances and evolution of *N*_*e*_ over time estimated using the three methods in Perendale sheep

**Fig. 6:**
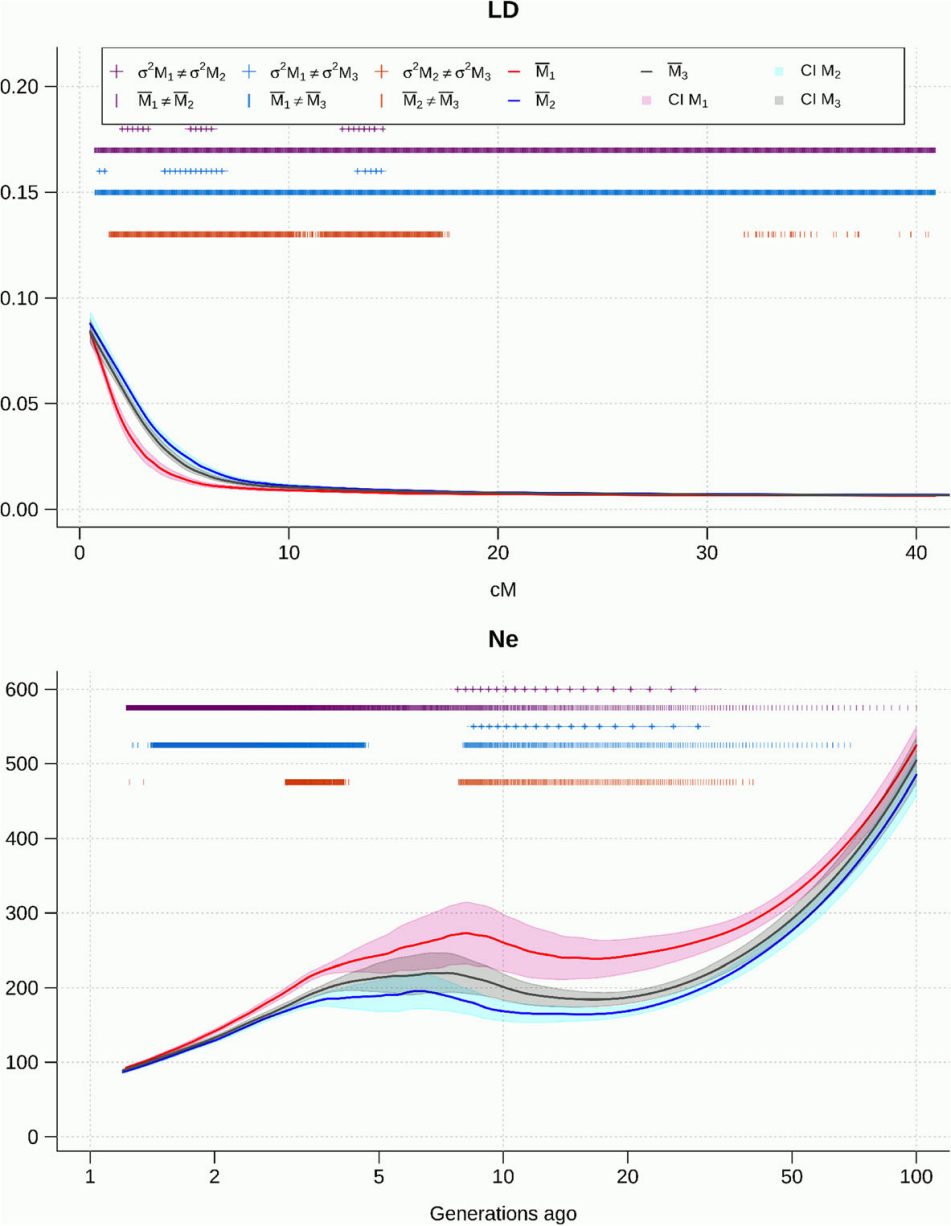
Linkage disequilibrium (LD) decay over genetic distances and evolution of *N*_*e*_ over time estimated using the three methods in Romney sheep

**Fig. 7:**
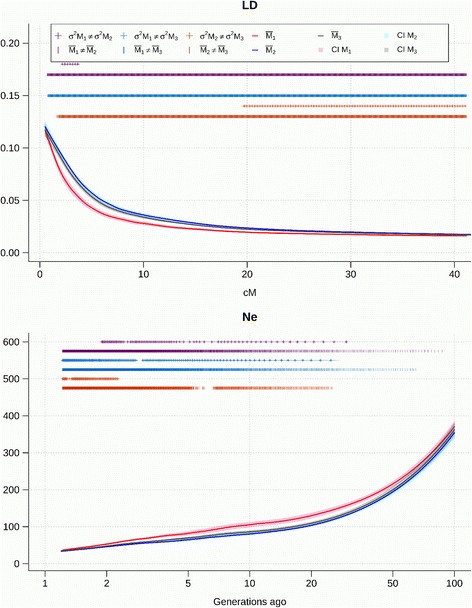
Linkage disequilibrium (LD) decay over genetic distances and evolution of *N*_*e*_ over time estimated using the three methods in Texel sheep

The LD decay and the evolution of *N*_*e*_ obtained using the three methods M1, M2, and M3 is shown in Figs 4, 5, 6 and 7 for the pure breeds, and in Additional files 7, 8 and 9 for the composite breeds. Curves were fitted as the mean of the 30 repetitions of a sample of 20,000 marker pairs for each of the 26 autosomes. The differences in means and variances between each of the three models were tested and significant differences at α = 0.05 are indicated on the graph. The 95% confidence interval is represented around each of the three curves. Effective population size was inferred from r^2^. The decline of N_e_ was estimated from one generation ago (50 cM between markers) up to 100 generations ago (0.5 cM between markers). In the Coopworth breed, significant differences were not observed between the variances for M1 and M2; and for M2 and M3 when investigating LD. However, variances for M1 were significantly larger than variances for M3, suggesting that M3 is more accurate than M1. This trend is also evident on *N*_*e*_ curves, where variances between M1 and M2 are significantly different. Most differences between LD and *N*_*e*_ averages observed using different methods were significant.

The results for the methods M2 and M3 were very similar, albeit significantly different for recent generations. The variances for these two methods were comparable. Using M1 increased the variability of estimates, accelerated the LD decay and produced estimates of *N*_*e*_ greater than using M2 or M3.

**Fig. 8:**
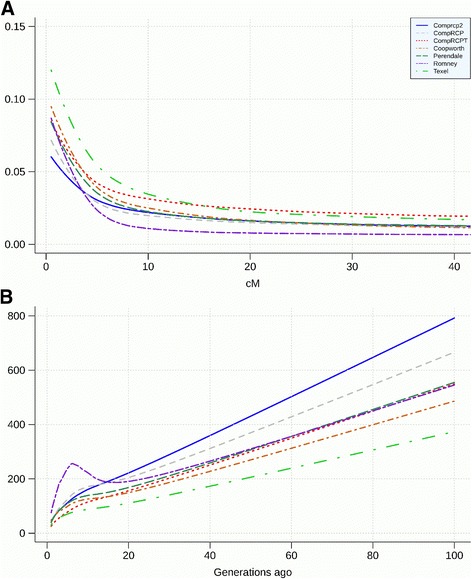
Comparison of **a**) Linkage Disequilibrium (LD) decay and **b**) *N*_e_ between breeds

The comparison between breeds for method M3 is shown in Fig. 8 and Table 4. There were significant differences between the different methods for LD and *N*_*e*_ estimates, but the ranking between breeds was not affected by the method used. The results of LD extent for Comprcp2 and CompRCP indicate a persistence of LD similar to that observed in Perendale and Coopworth. However, at short distances (smaller than 10 cM) the persistence of LD was lower than that observed in pure breeds. LD in Texel and CompRCPT persisted most over longer genetic distances.

**Table 4:** Estimation of historic effective population size for different intervals between one and 100 generations ago for Method 1 (M1) using 1 cM = 1 Mb, Method 2 (M2) using a chromosome-specific ratio of cM per Mb and method 3 (M3) using the expected genetic position per SNP

Breed denomination	Method	N_e_					Diff N_e_
		1	5	20	50	100	N_e_ 100 – N_e_ 1
Romney	M1	78	263^ab^	262^ab^	333^ab^	553	475
	M2	75	220^bc^	171^bc^	295^b^	525	450
	M3	77	237^ac^	188^ac^	305^a^	535	458
Coopworth	M1	50	119^ab^	185^ab^	293^ab^	506	456
	M2	46	95^bc^	139^bc^	260^b^	473	427
	M3	47	100^ac^	150^ac^	271^a^	489	442
Perendale	M1	41	130^ab^	210^ab^	332^ab^	573	532
	M2	39	106^bc^	159^b^	297^b^	541	502
	M3	40	111^ac^	170^a^	308^a^	555	515
Texel	M1	32	85^ab^	134^ab^	224^ab^	391	359
	M2	30	67^bc^	105^b^	202^b^	369	339
	M3	30	71^ac^	110^a^	205^a^	373	343
CompRCP	M1	45	139^a^	254^ab^	399^ab^	689	644
	M2	43	120^c^	187^bc^	349^b^	636	593
	M3	43	124^ac^	201^ac^	362^a^	652	609
CompRCPT	M1	27	86^ab^	183^ab^	315^ab^	556	529
	M2	26	76^bc^	148^b^	291^b^	537	511
	M3	26	78^ac^	155^a^	295^a^	539	513
Comprcp2	M1	42	130^ab^	267^ab^	460^ab^	811	769
	M2	41	111^bc^	212^bc^	415^b^	766	725
	M3	41	113^ac^	226^ac^	439^a^	806	765

^a,b,c^significant differences at α = 0.05 between N_e_ estimates in a breed for the same generation estimated using different methods

## Discussion

### Genetic diversity

This study was undertaken to understand the genetic structure and genetic variability of several sheep breed groups of economic importance in the NZ pastoral system. The estimates of genetic diversity indicated that the breeds present moderate to high levels of genetic diversity. Low values of inbreeding estimates were obtained using both genomic or pedigree data. Inbreeding estimates obtained from molecular markers are often greater than those obtained using pedigree information [31, 38]. However, in this study the estimates were quite similar and Auvray et al. [39] did not observe a significant difference between inbreeding coefficients based on genomic or pedigree information. The high average inbreeding estimate of 0.092 for Comprcp2 may be biased as the allele frequency used for the calculation of the genomic kinship matrix was predominantly from a breeding line developed by a private company from different specific breed groups of NZ sheep. In particular, their breeding line has an important genetic proportion of Dorset and Suffolk breeds (unpublished data), which were breeds not included in this study. The levels of heterozygosity were greater than those reported by Kijas et al. [4] (e.g. Romney: *H*_*e*_ = 0.314 vs. *H*_*e*_ = 0.358 in the present study and Texel: *H*_*e*_ = 0.296 vs. *H*_*e*_ = 0.354 in the present study) for NZ sheep, as well as for many others breeds from all around the world. The previous estimations were obtained using a much smaller set of animals (i.e. 18 for Romney and 12 for Texel), which were selected as 100% purebred animals, which could explain the lower *H*_*e*_ observed by Kijas et al. [4] compared to the results reported here.

The high number of polymorphic SNPs observed in this study is similar to estimates observed for a different and smaller dataset for a variety of worldwide breeds [4]. However, the SNPs selected to be included in the Illumina OvineSNP50 BeadChip have MAF greater than 0.25 and, therefore, ascertainment bias could have been introduced [4]. This potential ascertainment bias was previously investigated in Kijas et al. [40], who concluded that it is unlikely that estimates of genetic diversity between breeds and regions can be strongly modified by ascertainment bias, as a variety of breeds were used for SNP detection and SNP chip panel development.

### Relatedness between individuals and evidence for breed differentiation and admixture

The differentiation between breed groups and subgroups is mainly a consequence of animals coming from different farms with different genetic backgrounds and selection schemes. As mentioned previously, the two most distant breeds on the first MDS axis of Fig. 1a are Texel and Romney. The Romney breed arrived in NZ in 1880, while Texel was introduced only around 1990 [41]. Romney and Perendale appeared relatively genetically close to each other. This finding is consistent with the development history of these two breeds as Perendale is a composite breed formed by crossing Romney and Cheviot [41]. A great part of the development history of sheep breeds has been previously investigated using phylogenetic trees [3, 4, 40]. According to recorded history, Texel come from a Dutch Landrace crossed with Cheviot around 100 years ago. The composite breeds were scattered between pure breed clusters, as expected. The distribution of Comprcp2 animals in the single breed and overall MDS is consistent with the results observed on kinship and inbreeding estimation and reveals that this breed group is mainly composed of two subgroups, one made up of different proportions of the four pure breed groups, and the other one made up mainly of other breeds not included in this study. For instance, the tight blue cluster at the bottom right of Fig. 1a is composed largely of sheep with Dorset and Suffolk ancestry.

Crossbreeding is widely used in the NZ sheep industry to improve production efficiency in the wide range of climate and geographical conditions of the country [42]. However, some pure breeds remain genetically distinct according to the patterns observed. In addition to the genetic diversity between pure breeds, diversity within breed is also observed. Genetic diversity within breed is very important to allow selection and greater improvement in the traits of interest.

### Genetic map

In this study, the equation ${N}_e=\frac{1}{4 c}\left(\frac{1}{E\left({r}^2\right)}-1\right)$ was utilized, which is based on LD and estimated genetic distance between pairs of markers. An estimation of the genetic positions for the SNP, which is more accurate than the usual assumption that 1 cM = 1 Mb was determined. The results suggesting that recombination rates tend to be more variable on short chromosomes should be taken with caution. Due to the number of SNPs used to create the genetic maps, the low number of animals (~120) in the IMF and the curve smoothing procedure used to generate the graph, it is not possible to precisely identify the recombination “hot-spots”. A more informative mapping resource could be an option to improve these results.

In genetic analyses, it is common to assume that 1 Mb is equivalent to 1 cM [31]. However, the relationship between physical and genetic distances is very variable between and within species. For domesticated sheep no difference in genetic map size between males and females has been observed, contrary to the majority of mammalian species [43]. For wild Bighorn sheep the length of the autosomal female map was estimated at 3166 cM, while the length of the autosomal male map was estimate at 2831 cM [44]. Different genetic maps, obtained using various sets of SNPs or microsatellites are available for sheep. For instance, in Spanish Churra sheep [31], the genetic size of the genome was the highest value observed in sheep (4313 cM), which could be due to the version of the ovine genome used in that study. Furthermore, the highest value observed by Garcia-Gamez et al. [30] could be due to mapping or genotyping errors. Poor coverage of the genome by markers used to estimate the genome size can lead to underestimation, mainly if large numbers of double crossovers occur between adjacent markers or if the ends of the chromosomes are not covered. Overestimation could be due to the SNP order being occasionally incorrect on version 2 of the sheep genome assembly. In other words, if the order of two markers on the map is reversed or one is on another chromosome, it may artificially increase the genetic distance in that chromosomal region. In any case, results of Garcia-Gamez et al. [31] on the Spanish Churra sheep were quite different from other studies, such as an estimate of 3403 cM obtained using the International Mapping Flock [30] and an estimate of 3285 cM, calculated for Soay sheep [45]. Finally, the refined estimations of genome size used were comparable to the last map on the same flock (IMF, Table 3). However, due to a higher marker density, results using bi-allelic SNP markers are expected to be more accurate than a map designed from microsatellites [46].

### Estimations of extent of LD and ancestral effective population size

Hayes et al. [10] established the relation between genetic distance and the number of generations back related to the corresponding *N*_*e*_ estimate. Due to the inverse nature of this relationship, as shown by the function $T=\frac{1}{2 c}$, the number of SNP pairs available to investigate *N*_*e*_ in past generations decreases faster than the number of marker pairs per cM. This means that the estimate for 50 to 100 generations in the past is made on marker pairs at distances from 0.5 cM to 1 cM only.

Levels of LD varied between and within breeds, and according to which chromosome is considered and to the position of markers on a chromosome. The shapes of the LD and *N*_*e*_ curves for Perendale sheep are very close to those observed in Coopworth. However, interestingly, significant differences were not observed for variances of LD between M1 and M3 compared to those observed in Coopworth (Additional file 10). Despite this finding, significant differences for variances were observed at close distances between M1 and M2, and also between M1 and M3 for *N*_*e*_ which supports the conclusion that M2 and M3 improved the *N*_*e*_ estimation (Additional file 11). As in Coopworth, there were significant differences between the three methods, for mean LD and *N*_*e*_. *N*_*e*_ based on pedigree information was also calculated following the method described by Leroy et al. [47]. However, as shown in Additional file 1, the pedigree depth is too inconsistent and there were too many missing parents to estimate realistic estimates. In fact, there were cases where average inbreeding and relationship between animals for a generation was lower than the values for the previous generation, which led to negative N_e_ estimates. As these estimates have no biological interpretation, they were not presented here. This highlights another great advantage of using genomic data for genetic diversity analyses when there is missing pedigree information.

The greatest levels of LD at both short and long distances among all the pure breeds was observed for the Texel breed. Among the breeds included in this study, Texel was the most recently introduced breed in NZ. In addition, a restricted number of Texel animals were imported, which was reflected in the smallest ancestral *N*_*e*_ of the pure breeds. Unexpectedly, significant difference between M2 and M3 were found for mean LD at distances over 20 cM between markers. However, no difference was observed between the other methods for LD estimation.

The findings of this study suggest that the estimation of LD and *N*_*e*_ are significantly affected by the method used in the calculations. This is particularly apparent in estimations for the last 50 generations. However, probably due to the SNP density, *N*_*e*_ estimates for over 50 generations were not statistically significant. A similar trend was observed for the methods variability (represented on the various graphs by the 95% confidence interval around the expected values), even though significant differences between variances tend to disappear after approximately 20 to 30 generations in the past.

The differences between the three curves can only be observed when enough pairs of markers are available for each generation in the past. Therefore, to investigate *N*_*e*_ in a more distant past (i.e. greater than 100 generations ago) a denser SNP chip is required. However, the main emphasis of this study was to estimate relatively recent *N*_*e*_ (i.e., five to 10 generations ago). Investigating *N*_*e*_ for older events results in similar recombination rates for the three methods, and in turn leads to very little variation in estimation of *N*_*e*_ between the three methods. Higher SNP density is needed for estimating *N*_*e*_ in the very distant past.

On the other hand, for distances between markers greater than 10 cM (five generations ago or less), differences observed between the three methods for LD and *N*_*e*_ are small but usually remain significant. Similar results were observed for methods M2 and M3, albeit significantly different for recent generations. The variances between these two methods are comparable. At the same time, using M1 increases the variability of estimates, accelerates the LD decay and produces an estimate of *N*_*e*_ greater than using M2 or M3. To check whether the difference was due to using a constant ratio over the genome, or if the incorrect constant was used, a single overall estimator of the cM/Mb ratio for the whole genome was also investigated. This ratio resulted in similar results to M1. Method M2 estimated a more persistent LD across distances between markers compared to M3, and a lower *N*_*e*_. For M3, this seems to be a consequence of the variability of the recombination rate within a chromosome. Method M2 could be considered more robust, because it generates estimates that are less influenced by local mapping errors. Nevertheless, method M3 is assumed the most accurate measure with genetic distances to be the closest to the truth. Various quality control steps were also implemented to minimize the effects of mapping errors on the estimates. When genetically selecting for different traits in a breeding program, selection pressure may be imposed on different parts of the genome and in different intensities, depending on the genetic architecture of the trait and selection intensity for the trait. *N*_*e*_ is a useful parameter to measure the intensity of this selection as it provides insights regarding the evolution of the population history and genetic diversity. It can be estimated locally across the genome or averaged per chromosome or over the whole genome. Local estimation will require precise genetic positions for the markers for use with M3.

The large variability between chromosomes (represented by the confidence intervals around the curves of *r* ^2^ and N_e_) is mainly due to the overall recombination rate, which tended to increase as the size of the chromosome decreased. LD decay was mostly variable between the 26 chromosomes at distances lower than 10 cM. LD is greater at short distances for the smaller (acrocentric) chromosomes and decays rapidly, whereas for the metacentric chromosomes (OAR1, OAR2 and OAR3) LD appears lower at short distances, but is more persistent. The same results were observed for every chromosome and every breed in this study.

LD can be interpreted as a signature of past selection. For example, crossbreds would be expected to exhibit faster LD decay than purebreds and have lower LD at long distances. LD was utilized to explain the differences observed between breeds, where observed LD is a function of the recombination rate between loci within a breed and the selection performed for specific quantitative or qualitative traits of interest. The observed range of LD for Romney was expected, as breeders have aimed to maintain a large genetic diversity since the development of the breed around 130 years ago. A similar trend was observed for Coopworth and Perendale, however, to a smaller degree. The persistence of LD over long genetic distances for CompRCPT and Texel was expected given the small number of flocks, which might have created a bottleneck [41], recent crossbreeding and also due to the contributions from two specific sub-populations of Texel, and East Friesian. Texel sheep presents the most persistent LD over time and the smallest *N*_*e*_. The range of LD and the evolution of *N*_*e*_ characterize the degree of genetic diversity per breed. For all breeds, a dramatic decrease of recent *N*_*e*_ is observed. Romney sheep seems to have been better managed in terms of mating and preservation of diversity prior to the wide utilisation of AI. Despite *N*_*e*_ variations observed within each chromosome and between all chromosomes, estimated *N*_*e*_ values averaged over all chromosomes should be close to the true *N*_*e*_ given that the genotyped animals represent the population of interest and assuming a similar level of selection over each chromosome.

The particular shape of the *N*_*e*_ curve for Romney (Fig. 6) reflects its history and reveals a decrease of *N*_*e*_ from 100 generations until 20 generations ago, followed by an increase until six generations ago before a sharp fall. Romney were imported to NZ, about 100 to 130 years ago and after a strong period of expansion in numbers for over a century due to successful management, *N*_*e*_ has decreased dramatically in the last 10 to 20 years. This occurred when laparoscopic artificial insemination (AI) in ram breeding flocks started to be used more widely, in association with the decrease of the NZ sheep population and greater selection intensity.

With M1, genetic positions for the markers were considered proportional to the physical positions, using the same ratio of 1 cM/Mb over the whole genome. This first estimation of LD decay over distance and *N*_*e*_ estimation over time showed a large variability between chromosomes and the reason for this variability was not clear. Consequently, it was decided to estimate the genetic size of the sheep chromosomes, and to create a new genetic map (Additional file 5) for 6629 SNP markers. This map was used to re-estimate LD and *N*_*e*_ (M2 and M3). The results show clearly that using M2 or M3 affects the estimates of LD and *N*_*e*_ compared to M1. LD appears always more persistent using M2 or M3 and *N*_*e*_ was significantly lower from the present time to about 60 generations in the past. The reduction of the confidence interval around the expected *r* ^2^ and *N*_*e*_, which indicates a reduction of the variance of *r* ^2^ and *N*_*e*_ between the 26 chromosomes, shows improvement in terms of accuracy using M2 and M3 in particular. The reduction of the confidence interval is mainly observed for the closest markers separated by less than five cM. For *N*_*e*_, the variance between chromosomes is more reduced for values between one and 20 past generations, which correspond to markers separated by 2.5 cM or more.

The size of the samples used for the calculations varied, which could impact the calculations of LD and *N*_*e*_. Weir and Hill [48] suggested a correction for sample size. However, other studies have not found significant differences when doing these adjustments (e.g., [12]). In this study, we observed that the differences would also be small (i.e., maximum increase of 3 to 15% in *N*_*e*_ for the small sample size populations from many to a few generations ago). Other studies have reported estimates of *N*_*e*_ for different sheep breed populations. For instance, Brito et al. [42], reported estimates of *N*_*e*_ for New Zealand terminal sire composite breeds ranging from 125 (Dual-Purpose) to 974 (Primera), which are higher than the estimates obtained in this study and reflect the greater genetic diversity of those populations. Zhao et al. [49] reported *N*_*e*_ estimates at seven generations ago ranging from 67 to 207, which are more similar to the estimates from this study. Absolute values of *N*_*e*_ varied between breeds but trends were very similar. Those estimates can be compared with the recommendation of the FAO [50], suggesting a minimum *N*_*e*_ of 50 to maximise response to selection and to guarantee that a positive genetic trend can be maintained. This leads to a rate of inbreeding of 1% per generation. In practice, an effective number of 100 animals per generation is suggested for sheep [38]. In any domestic species where a strong selection is applied, a drop in *N*_*e*_ is always observed. This was also observed in this study where *N*_*e*_ decreased linearly from 50 generations in the past up to five generations in the past. The decay of *N*_*e*_ over the last 15 years (about five generations) is probably due to the wider use of AI, advanced genetic evaluation techniques (e.g. BLUP), as well as larger progeny groups for elite sires and a reduction in recorded stud ewe numbers. Texel have been recently introduced in NZ, and the national population is derived from about 200 animals and 30 rams [51]. In any case, the current *N*_*e*_ estimated in this study for each breed is always lower than the *N*_*e*_ estimates observed in Churra, which reach 128. Likewise, recent estimates of *N*_*e*_ in many breeds [41] are greater than those estimated in the populations included in this study. The *N*_*e*_ estimates might be improved with more animals sampled, and using stricter breed definitions could also be beneficial. If the very low estimates of *N*_*e*_ at one generation ago reported in Table 4 are confirmed, it indicates that some NZ sheep breeds may be too close genetically (within breeds) to maintain good fitness for long term selection. But even if the threshold recommended by the FAO is set as 50, in practice, this threshold is often crossed. However, animal breeders could introduce new germplasm into their flocks to avoid potential issues due to the dramatic reduction in *N*_*e*_ such as what has been reported in some cattle breeds where breed viability is threatened [52]. To conclude, the genetic diversity of the NZ sheep breeds studied remains acceptable apart from Texel, but the important drop of *N*_*e*_ observed should motivate breeders to try to alleviate a further decrease before problems resulting in a lack of genetic variability occur. Furthermore, this method is robust and not time consuming compared to the other methods (results not shown). This study will be complemented by a second investigation focused on signatures of selection using local *r* ^2^, *N*_*e*_ and Fst [53, 54]. Next generation sequencing (NGS) data may also be important for the study of LD in sheep. For instance, Yang et al. [55] reported population structure and genetic diversity of sheep breeds using NGS data.

## Conclusions

Our findings indicate that using M2 or M3 affects the estimates of LD and *N*_*e*_ compared to M1. Linkage disequilibrium appears to be more persistent using M2 or M3 and *N*_*e*_ was significantly lower from the current generation to 60 generations in the past. Using M1 leads to an overestimation of *N*_*e*_, especially for recent times. This can be problematic if *N*_*e*_ is used to inform inbreeding control practices and selection intensity. However, differences observed are not important if *N*_*e*_ is used to characterize population history. Using a denser SNP chip and an estimation of the genetic distances based on a population with more than three generations of animals genotyped would improve the estimates and could help locate recombination “hot-spots”.

Our results indicate that the effective population sizes of these breeds have been declining. This is important for the New Zealand sheep industry and there should be efforts to reduce this decline, such as selection decisions that balance genetic gain against genetic diversity.

Finally, the methods investigated here can be applied to investigate the evolution of genetic diversity in any species that reproduces sexually. The differences observed between the methods are clearer if the density of markers is sufficient (~ > 1 marker/50 Kb). The same study performed on a denser SNP chip panel, such as the ISGC Illumina Ovine HD BeadChip (606 K), would allow checking the differences between our methods for the estimates of LD at very short distance and *N*_*e*_ at more than 100 generations in the past.

## Additional files

Additional file 1: Description of the population based on pedigree information. The description of the populations based on pedigree information is given. (XLSX 15 kb)

Additional file 2: Workflow Summary. Description of the processes established to perform quality control and data analysis. The workflow per breed leads to the analysis of the three methods tested in this paper to calculate LD and *N*_*e*_ within breed. The workflow applied to the full data set allowed us to estimate the genetic distances between breed. This will allow investigation of signatures of selection in the sheep genome using local Fst, LD and *N*_*e*_ across the genome. (TIFF 162 kb)

Additional file 3: Description of the markers used for the analyses including physical position, number of informative meiosis, number of meiosis with known phase. (XLSX 320 kb)

Additional file 4: OvineExpectedRecombinationRate.txt. This file contains information about physical and expected recombination rate for the entire sheep genome assembly (ISGC Oar_v3.1). The first line gives the correct setting to load it in the UCSC genome browser. This file contains four columns, the chromosome, the physical position for the previous markers, the physical position for the markers, and the expected recombination rate between the two markers. (TXT 1492 kb)

Additional file 5: OvineGeneticMap.IMF.txt. This file contains information about physical and genetics positions for the entire sheep genome assembly (ISGC Oar_v3.1). The first line gives the correct setting to load it in the UCSC genome browser. This file contains four columns, the chromosome, and the physical position for the previous markers, the physical position for the markers, and the genetic position for a marker. (TXT 1492 kb)

Additional file 6: Average number of marker pairs available for *N*_*e*_ estimation, per generation. The hyperbolic nature of the equation used to define the relationship between genetic distances between markers and number of generations in the past explains the fast decrease of the number of marker pairs available per generation. (TIFF 119 kb)

Additional file 7: LD decay over genetic distances and evolution of *N*_*e*_ over time estimated using the three methods in Comprcp2 sheep. At short distances, the Comprcp2 sheep gave the lowest LD (0.06) and, at 15 generations ago, the highest estimate of *N*_*e*_ of the breed groups studied. This could be due to this population being derived from different genetically distant breed groups such as Romney, Coopworth and Perendale but also Dorset and Suffolk. The tests on variances show that almost no significant differences were observed between methods except between M1 and M2, and M1 and M3 when estimating *N*_*e*_. Hypothesis testing on means shows however that the differences between each of the three methods are significant for both LD and *N*_*e*_, for M1 and M2, and M1 and M3. Differences between M2 and M3 are less significant. (TIFF 120 kb)

Additional file 8: LD decay over genetic distances and evolution of *N*_*e*_ over time estimated using the three methods in CompRCP sheep. The LD curve for the CompRCP breed is similar to those observed for Comprcp2, but present a higher LD at short distances and a relatively high *N*_*e*_ after 15 generations. Knowing the breed composition of CompRCP, the results are consistent with what would be expected; i.e. a lower LD at short distances but very persistent, and a greater *N*_*e*_ than that observed in pure breeds. The tests of variances show almost no significant differences between methods except for the *N*_*e*_ estimation between M1 and M2, and M1 and M3. Test of means show, however, that the differences between each of the three methods are almost always significant for both LD and *N*_*e*_. (TIFF 117 kb)

Additional file 9: LD decay over genetic distances and evolution of *N*_*e*_ over time estimated using the three methods in CompRCPT sheep. The curves of LD for the CompRCPT are slightly different to those observed for the other composites. Indeed, LD appears comparatively high at short distances, decreases faster as the distance between markers increases, but stays the greatest at long distances compared to any other breed. At the same time, this breed shows the lowest *N*_*e*_ at one generation in the past, while at 100 generations ago the LD and *N*_*e*_ curves were similar to those observed in Texel. This is likely due to the proportion of Texel in this group. (DOCX 16 kb)

Additional file 10: Mean and variance r^2^ estimated for each chromosome and for 2000 genetic distance points evenly distributed between 0.05 and 50 cM (100 and 1 generations in the past, respectively). For instance, the spreadsheets for the mean r^2^ were coded as: meanR2rep1_CompRCPT.xlsx, where 1 indicates method 1 and “CompRCPT” indicates the corresponding breed group. The spreadsheets for the r^2^ variances were coded as: “varR2rep1_CompRCPT.xlsx”, for example. (ZIP 29639 kb)

Additional file 11: Mean and variance *N*_*e*_ estimated for each chromosome and for 2000 genetic distance points evenly distributed between 0.05 and 50 cM (100 and 1 generations in the past, respectively). The spreadsheets for the mean N_e_ were coded as: meanNerep1_CompRCPT.xlsx, where 1 indicates method 1 and “CompRCPT” indicates the breed group. The spreadsheets for the N_e_ variances were coded as: “varNerep1_CompRCPT.xlsx”, for example. (ZIP 24908 kb)

## Abbreviations

CM: centiMorgan
Coop: Coopworth
GRM: Genomic relationship matrix
*H*_*e*_: Average heterozygosity
HWE: Hardy Weinberg Equilibrium
IBS: **I**dentity by state
LD: Linkage disequilibrium
MAF: Minor allele frequency
Mb: Mega base pairs
MDS: Multi-dimensional scaling
N_e_: Effective population size
NZ: New Zealand
Peren: Perendale
QC: Quality control
Rom: Romney
SIL: Sheep Improvement Limited
SNP: Single Nucleotide Polymorphisms
Tex: Texel
VNTR: Variable Number Tandem Repeats
