# Supplementary figures and images for: Estimation of linkage disequilibrium and effective population size in New Zealand sheep using three different methods to create genetic maps
Source: BMC Genet. 2017 Jul 21;18:68. doi: 10.1186/s12863-017-0534-2 (PMC5521107; doi:10.1186/s12863-017-0534-2)

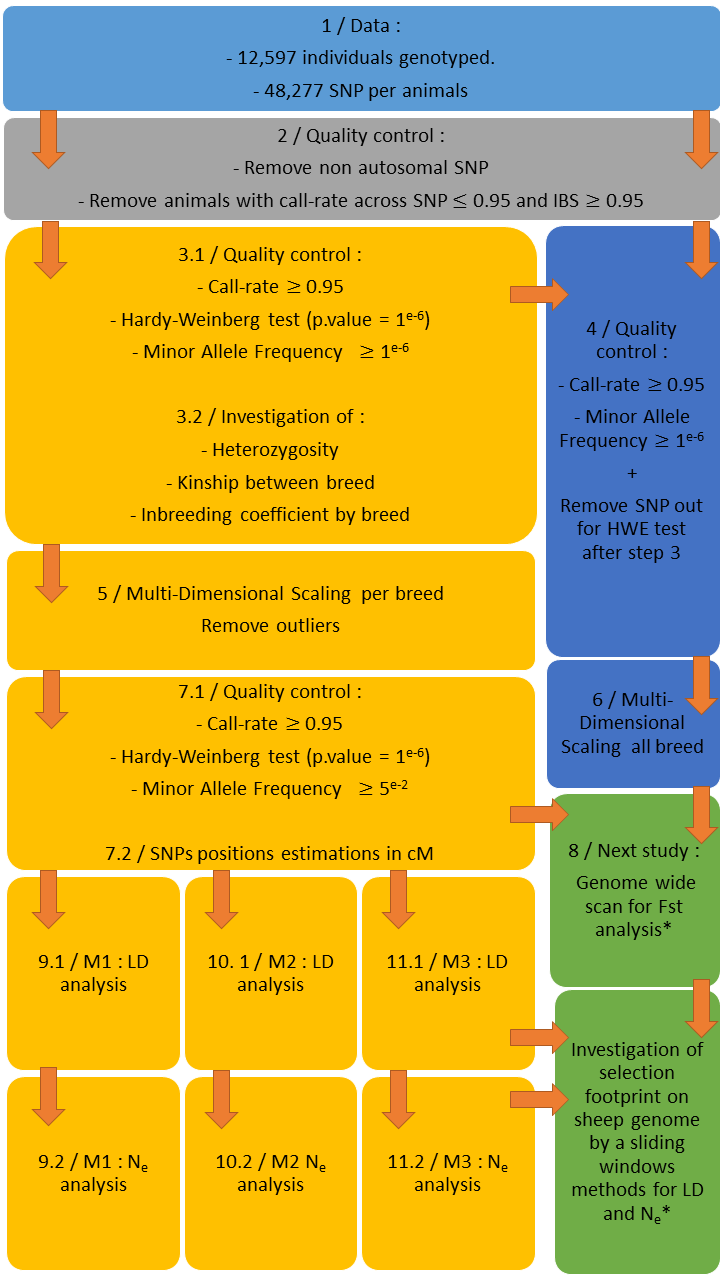

Supplement: Supplementary file 2 — Workflow Summary. Description of the processes established to perform quality control and data analysis. The workflow per breed leads to the analysis of the three methods tested in this paper to calculate LD and N e within breed. The workflow applied to the full data set allowed us to estimate the genetic distances between breed. This will allow investigation of signatures of selection in the sheep genome using local Fst, LD and N e across the genome. (TIFF 162 kb) [file 12863_2017_534_MOESM2_ESM.tif]
