# Supplementary material for: Estimation of linkage disequilibrium and effective population size in New Zealand sheep using three different methods to create genetic maps
Source: BMC Genet. 2017 Jul 21;18:68. doi: 10.1186/s12863-017-0534-2 (PMC5521107; doi:10.1186/s12863-017-0534-2)
Supplement: Supplementary file 9 — LD decay over genetic distances and evolution of N e over time estimated using the three methods in CompRCPT sheep. The curves of LD for the CompRCPT are slightly different to those observed for the other composites. Indeed, LD appears comparatively high at short distances, decreases faster as the distance between markers increases, but stays the greatest at long distances compared to any other breed. At the same time, this breed shows the lowest N e at one generation in the past, while at 100 generations ago the LD and N e curves were similar to those observed in Texel. This is likely due to the proportion of Texel in this group. (DOCX 16 kb) [file 12863_2017_534_MOESM9_ESM.docx]

| **Table S8.** Average number of marker pairs available for N_e_ estimation, per generation. | | | | | | | |
| --- | --- | --- | --- | --- | --- | --- | --- |
| **Gen. ago** | **Number of marker pairs** |  | **Gen. ago** | **Number of marker pairs** |  | **Gen. ago** | **Number of marker pairs** |
| [99;100] | 3,426 |  | [66;67] | 7,593 |  | [33;34] | 28,703 |
| [98;99] | 3,477 |  | [65;66] | 7,831 |  | [32;33] | 30,648 |
| [97;98] | 3,571 |  | [64;65] | 8,191 |  | [31;32] | 32,510 |
| [96;97] | 3,579 |  | [63;64] | 8,300 |  | [30;31] | 34,604 |
| [95;96] | 3,847 |  | [62;63] | 8,706 |  | [29;30] | 36,837 |
| [94;95] | 3,757 |  | [61;62] | 8,882 |  | [28;29] | 39,409 |
| [93;94] | 3,961 |  | [60;61] | 9,140 |  | [27;28] | 42,032 |
| [92;93] | 3,956 |  | [59;60] | 9,469 |  | [26;27] | 45,335 |
| [91;92] | 4,113 |  | [58;59] | 9,778 |  | [25;26] | 48,785 |
| [90;91] | 4,070 |  | [57;58] | 10,130 |  | [24;25] | 52,535 |
| [89;90] | 4,265 |  | [56;57] | 10,575 |  | [23;24] | 57,194 |
| [88;89] | 4,335 |  | [55;56] | 10,850 |  | [22;23] | 62,099 |
| [87;88] | 4,491 |  | [54;55] | 11,298 |  | [21;22] | 67,707 |
| [86;87] | 4,479 |  | [53;54] | 11,539 |  | [20;21] | 74,143 |
| [85;86] | 4,561 |  | [52;53] | 12,242 |  | [19;20] | 81,665 |
| [84;85] | 4,771 |  | [51;52] | 12,480 |  | [18;19] | 90,016 |
| [83;84] | 4,964 |  | [50;51] | 13,151 |  | [17;18] | 100,292 |
| [82;83] | 4997 |  | [49;50] | 13,561 |  | [16;17] | 112,125 |
| [81;82] | 5,173 |  | [48;49] | 14,094 |  | [15;16] | 126,099 |
| [80;81] | 5,203 |  | [47;48] | 14,744 |  | [14;15] | 143,019 |
| [79;80] | 5,339 |  | [46;47] | 15,195 |  | [13;14] | 164,356 |
| [78;79] | 5,559 |  | [45;46] | 16,000 |  | [12;13] | 190,081 |
| [77;78] | 5,617 |  | [44;45] | 16,746 |  | [11;12] | 223,210 |
| [76;77] | 5,795 |  | [43;44] | 17,333 |  | [10;11] | 266,424 |
| [75;76] | 5,950 |  | [42;43] | 18,225 |  | [9;10] | 323,035 |
| [74;75] | 6,098 |  | [41;42] | 19,089 |  | [8;9] | 398,500 |
| [73;74] | 6,340 |  | [40;41] | 20,046 |  | [7;8] | 507,875 |
| [72;73] | 6,395 |  | [39;40] | 20,917 |  | [6;7] | 667,047 |
| [71;72] | 6,633 |  | [38;39] | 22,191 |  | [5;6] | 919,880 |
| [70;71] | 6,758 |  | [37;38] | 23,052 |  | [4;5] | 1,349,077 |
| [69;70] | 6,992 |  | [36;37] | 24,336 |  | [3;4] | 2,213,412 |
| [68;69] | 7,408 |  | [35;36] | 25,717 |  | [2;3] | 4,275,764 |
| [67;68] | 7,352 |  | [34;35] | 27,358 |  | [1;2] | 11,181,609 |
| **Gen.:** generation | | | | | | | |
